# P-1479. Patterns of surgical antibiotic prophylaxis among patients undergoing Cesarean deliveries in tertiary care hospitals in Bangladesh

**DOI:** 10.1093/ofid/ofae631.1649

**Published:** 2025-01-29

**Authors:** Md Golam Dostogir Harun, Md Shariful Amin Sumon, Firdausi Qadri, Robir Kumar Ghosh, Aninda Rahman, Syed Abul Hassan Md Abdullah, Md Saiful Islam, Thomas G Weiser, Lisa P Oakley, Ashley R Styczynski, S Cornelia Kaydos-Daniels

**Affiliations:** icddrb, Dhaka, Dhaka, Bangladesh; icddr,b, Dhaka, Dhaka, Bangladesh; International Centre for Diarrhoeal Disease Research, Bangladesh, Dhaka - , Bangladesh; icddr,b, Dhaka, Dhaka, Bangladesh; Directorate General of Health Services, Government of Bangladesh., Dhaka, Dhaka, Bangladesh; Bangladesh University of Professionals, Dhaka, Dhaka, Bangladesh; University of New South Wales, Sydney, South Australia, Australia; Stanford University Medical Center, Stanford, California; Centers for Disease Control and Prevention, Atlanta, GA, United States, Atlanta, Georgia; Centers for Disease Control and Prevention, Atlanta, GA; CDC Bangladesh Country Office, Dhaka, Dhaka, Bangladesh

## Abstract

**Background:**

The World Health Organization recommends a single dose of a first-generation cephalosporin given 30-60 minutes prior to surgery to prevent surgical site infections (SSI) in Cesarean deliveries (C-section). Perinatal exposure to antibiotics may modulate the microbiome of mothers and newborns, favoring the persistence of drug-resistant organisms which are frequently implicated in neonatal sepsis. This study investigated the pattern of surgical antibiotic prophylaxis (SAP) among patients undergoing C-sections in Bangladeshi tertiary care hospitals

Commonly prescribed antibiotics to surgery patients

**Figure:**
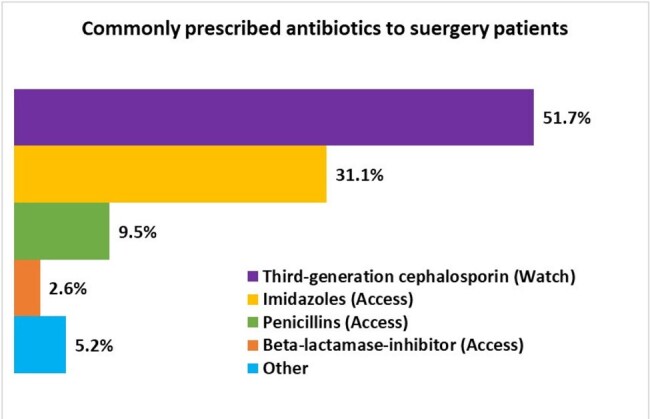

Commonly prescribed antibiotics to surgery patients

**Methods:**

From May through December 2023, we conducted a prospective observational study in six tertiary care hospitals and enrolled 1,335 women who underwent C-sections. Information on demographics and types of prophylactic antibiotic use was collected during the preoperative, postoperative, and post-discharge phases

**Results:**

The median age of enrolled participants was 26 years (IQR: 22-28) and nearly half (48.9%) had a prior C-section. Pre-operatively, ceftriaxone was the most common SAP, which was used in 88% of cases. The median time for pre-operative antibiotic administration until incision was 180 minutes (IQR: 90-270). Post-operatively, injectable antibiotics were administered to all patients, with ceftriaxone (92.6%) and metronidazole (95.3%) being the most frequently prescribed. Similarly, all patients received oral antibiotics upon discharge. Cefixime (84.6%) and metronidazole (51.0%) were the most prescribed antibiotics at hospital discharge, followed by flucloxacillin (48.4%) and amoxicillin-clavulanic acid (13.1%), for a median duration of 5 days

**Conclusion:**

Patients undergoing C-sections were frequently prescribed broad-spectrum antibiotics as part of SAP with extended post-operative dosing. Extensive SAP use may suggest underlying concerns regarding patient population, local antimicrobial resistance profiles, infection prevention lapses, and a lack of local hospital-based SAP guidelines. Understanding motivations for and effects of SAP patterns is essential for mitigating unnecessary perinatal antibiotic exposure and guiding antimicrobial stewardship efforts. Additionally, the timing of pre-operative SAP administration warrants further optimization

**Disclosures:**

**All Authors**: No reported disclosures

